# Circulating cathepsin-S levels correlate with GFR decline and sTNFR1 and sTNFR2 levels in mice and humans

**DOI:** 10.1038/srep43538

**Published:** 2017-02-27

**Authors:** Dominik Steubl, Santhosh V. Kumar, Maia Tato, Shrikant R. Mulay, Anders Larsson, Lars Lind, Ulf Risérus, Lutz Renders, Uwe Heemann, Axel C. Carlsson, Johan Ärnlöv, Hans-Joachim Anders

**Affiliations:** 1 Abteilung für Nephrologie, Klinikum rechts der Isar, Technische Universität München, Munich, Germany; 2 Medizinische Klinik und Poliklinik IV, Renal Division, Klinikum der Universität München, Campus Innenstadt, München, Germany; 3 Department of Medical Sciences, Uppsala University, Uppsala, Sweden; 4 Departments of Public Health and Caring Sciences/Clinical Nutrition, Uppsala University, Uppsala, Sweden; 5 Division of Family Medicine, Department of Neurobiology, Care Sciences and Society, Karolinska Institutet, Huddinge, Sweden; 6 School of Health and Social Studies, Dalarna University, Falun, Sweden

## Abstract

Cardiovascular complications determine morbidity/mortality in chronic kidney disease (CKD). We hypothesized that progressive CKD drives the release of cathepsin-S (Cat-S), a cysteine protease that promotes endothelial dysfunction and cardiovascular complications. Therefore, Cat-S, soluble tumor-necrosis-factor receptor (sTNFR) 1/2 and glomerular filtration rate (GFR) were measured in a CKD mouse model, a German CKD-cohort (MCKD, n = 421) and two Swedish community-based cohorts (ULSAM, n = 764 and PIVUS, n = 804). Association between Cat-S and sTNFR1/2/GFR was assessed using multivariable linear regression. In the mouse model, Cat-S and sTNFR1/2 concentrations were increased following the progressive decline of GFR, showing a strong correlation between Cat-S and GFR (r = −0.746, p < 0.001) and Cat-S and sTNFR1/sTNFR2 (r = 0.837/0.916, p < 0.001, respectively). In the human cohorts, an increase of one standard deviation of estimated GFR was associated with a decrease of 1.008 ng/ml (95%-confidence interval (95%-CI) −1.576–(−0.439), p < 0.001) in Cat-S levels in MCKD; in ULSAM and PIVUS, results were similar. In all three cohorts, Cat-S and sTNFR1/sTNFR2 levels were associated in multivariable linear regression (p < 0.001). In conclusion, as GFR declines Cat-S and markers of inflammation-related endothelial dysfunction increase. The present data indicating that Cat-S activity increases with CKD progression suggest that Cat-S might be a therapeutic target to prevent cardiovascular complications in CKD.

Cathepsin-S (Cat-S) is a cysteine protease with intracellular and extracellular proteolytic activities^1^. Within endosomes, Cat-S is involved in the processing of the major histocompatibility class II complex during antigen presentation, an essential element of host defense and autoimmunity^2^,^3^. In inflammatory states, Cat-S is released by activated monocytes and neutrophils into the circulation where it contributes globally to endothelial dysfunction by activating protease-activated receptor (PAR)-2 on microvascular endothelial cells to increase microvascular permeability and leukocyte adhesion^4^,^5^. Furthermore, release of Cat-S by activated macrophages within the vascular wall degrades the elastic fibers of the tunica elastic, a process that contributes to vascular wall degeneration, media calcification, and aneurysm formation^6^,^7^,^8^,^9^,^10^,^11^,^12^. Indeed, circulating Cat-S levels have been identified as a biomarker for age-related vascular diseases, hypertension, obesity, diabetes, and overall mortality in large population-based cohort studies^13^,^14^,^15^,^16^,^17^,^18^. Recent data from mouse models suggest that selective inhibition of Cat-S can attenuate renal inflammation in lupus nephritis and reduce atherosclerosis in CKD^2^,^19^. Therefore Cat-S might be a therapeutic target in proinflammatory states, such as arterio-/atherosclerosis.

Chronic kidney disease (CKD) is a systemic inflammatory state that dramatically accelerates cardiovascular morbidity and mortality^20^,^21^. The causal relationships between uremia, systemic inflammation and cardiovascular disease in CKD are not entirely clear. Based on the findings mentioned above, we hypothesized that CKD and systemic inflammation are associated with increasing levels of circulating Cat-S. To test this hypothesis, in a mouse model of progressive CKD and in three clinical cohorts of CKD patients we quantified glomerular filtration rate (GFR) as a measure of CKD, serum levels of soluble tumor necrosis factor-alpha receptors (sTNFR1 and sTNFR2) as a measure of systemic inflammation, and serum/plasma concentrations of Cat-S.

## Materials and Methods

### Animal studies

All experiments were performed in accordance with relevant guidelines and regulations and were approved by the regional Government of Oberbayern, Germany. Male C57BL/6 mice were obtained from Charles River (Sulzfeld, Germany) at the age of 7–8 weeks, mice were housed in groups of five in standard housing conditions with unlimited access to food and water for one week for acclimatization. CKD was induced by feeding an oxalate-rich diet, which was prepared by adding 50 μmol/g sodium oxalate to a calcium-free standard diet (Ssniff, Soest, Germany) as previously described^22^. Oxalate-rich diet was fed for 21 days. This model simulates progressive CKD and its complications such as metabolic acidosis. Plasma samples were collected prior to diet change and at days 1, 7, 14, and 21. All experimental procedures were approved by the local government authorities. All plasma samples were analyzed for the levels of Cat-S, sTNFR1, and sTNFR2 using commercially available enzyme-linked immunosorbent assay (ELISA) kits (Cloud-clone Corp. TX, USA, SEB933Mu; Sino Biologicals Inc. PA, SEK50496 and SEK50128, respectively).

### Glomerular filtration rate measurement in conscious mice

Glomerular filtration rate (GFR) was measured at different time points (day 0, 1, 7, 14 and 21) using an automated miniaturized imager device (Medibeacon, Germany). The mouse neck was shaved and the device was mounted on the shaved area using double-sided adhesive tape, then fluorescein-isothiocyanate(FITC)-sinistrin (Roche, Mannheim, Germany) was injected intravenously to each mouse at the dose of 150 mg/kg. The transcutaneous FITC signal was recorded continuously for a period of 1.5 h. Imaging data were analyzed using MPD Lab software (Medibeacon) and GFR was calculated from the decrease of fluorescence intensity over time using a two-compartment calculation algorithm as described^23^.

### Human cohorts and definition of chronic kidney disease stages

The Munich CKD cohort (MCKD) consisted of 421 patients from the outpatient clinic at Klinikum rechts der Isar, Munich, Germany. Among them were 69 patients without kidney disease (CKD 0°) serving as controls and 352 patients normally distributed across stages I°-V° 24 of CKD. All participants provided written informed consent. The study adhered to the declaration of Helsinki and was approved by the local ethics committee.

The Uppsala Longitudinal Study of Adult Men (ULSAM) was initiated in 1970. All 50-year-old men born between 1920 and 1924 and living in Uppsala, Sweden, were invited to participate in a health survey aimed at identifying cardiovascular risk factors (13, described in detail at http://www.pubcare.uu.se/ULSAM). The analyses presented in this study are based on the fourth examination cycle of ULSAM, in which participants were approximately 77 years old (1998–2001). Of 1398 invited men, 838 (60%) participated. Of these, 89 were excluded due to missing data on Cat-S, GFR or co-variates, leaving 749 participants as the present study sample. All 70-year-old individuals living in Uppsala, Sweden, between 2001 and 2004 were eligible for the Prospective Investigation of the Vasculature in Uppsala Seniors (PIVUS) study (13, described in detail at http://www.medsci.uu.se/pivus/pivus.htm). In the present study the second examination cycle of PIVUS was used (2006–2009), in which participants were 75 years old. Of 964 invited participants, 827 participated (86%). Of these, 23 participants were excluded due to missing data on Cat-S, GFR or co-variates, leaving 804 participants as the present study sample. All participants gave written informed consent and the ethics committee of Uppsala University approved both study protocols. The studies adhere to the declaration of Helsinki.

We applied the criteria for CKD stages according to the current Kidney Disease: Improving Global Outcomes (KDIGO) guidelines^24^: “CKD is defined as abnormalities of kidney structure or function, present for >3 months, with implications for health.” Therefore, the diagnosis of CKD was made when either estimated glomerular filtration rate (eGFR) was <60 ml/min/1.73 m^2^ of body surface area (BSA) and/or apparent signs of kidney damage were present. For diagnosis of kidney damage, we defined proteinuria with a cut-off >150 mg/g creatinine (MCKD) or albuminuria >26.4 mg/g creatinine (ULSAM and PIVUS) on spot-urine specimen and/or histologically proven kidney disease and/or abnormalities detected in imaging techniques (ultrasound, computed tomography, magnetic resonance imaging or nuclear imaging). Once the diagnosis of CKD was established, patients were assigned to a certain stage of CKD according to the KDIGO recommendation: CKD I° when eGFR >90 ml/min/1.73 m^2^ BSA, CKD II° 61–90 ml/min/1.73 m^2^ BSA, CKD 3° 31–60 ml/min/1.73 m^2^ BSA, CKD IV° 16–30 ml/min/1.73 m^2^ BSA and CKD V° 0–15 ml/min/1.73 m^2^ BSA. Patients with a single kidney (e.g. due to nephrectomy) were assumed to have CKD despite an eGFR >60 ml/min/1.73 m^2^ BSA. 69 patients in MCKD without a medical history for CKD and an eGFR >60 ml/min/1.73 m^2^ BSA were assigned to the group CKD 0°. Patients’ demographics of MCKD and PIVUS/ULSAM are given in Table 1, 2 and 3. Standard methods were used to assess anthropometrical measurements, blood pressure, and blood sampling. Venous blood samples were drawn in the morning after an overnight fast and stored at −80 °C.

### Biomarker analyses

For biomarker analysis, plasma was used in MCKD and serum in PIVUS/ULSAM. Creatinine and blood-urea-nitrogen (BUN) were measured using photometric techniques (creatinine: normal range 0.7–1.3 mg/dl in males and 0.5–1.1 mg/dl in females; BUN: normal range 7–18 mg/dl). In MCKD, cystatin C levels were assessed using a nephelometric immunoassay (normal range 0,50–0,96 mg/l); or using a latex-enhanced reagent (NLatexCystatin C; Siemens, Deerfield, IL, USA) detected using a BN ProSpec analyser (Siemens) in ULSAM; or with latex-enhanced reagents (Gentian, Moss, Norway) detected using an Architect ci8200 (Abbott Laboratories, Abbott Park, IL, USA) in PIVUS. In MCKD, the eGFR equation was calculated based on serum creatinine and cystatin C concentrations, as published^25^. In ULSAM and PIVUS, eGFR was assessed by a sole cystatin C-equations, as no data on circulating creatinine was available in these cohorts^26^. High-sensitive CRP measurements were performed using a latex-enhanced reagent (Siemens) assayed on a BN ProSpec^®^ analyzer (Siemens) in ULSAM; with the use of an Architect ci8200 in PIVUS; or using a turbidimetric measurement in MCKD. Serum levels of Cat-S were measured by an ELISA (human cathepsin S [total] DY1183; R&D Systems, Minneapolis, Minnesota). In the MCKD cohort, blood samples were frozen for a median of 515 days (range 340–677 days) until Cat-S levels were quantified in November 2015. In the ULSAM cohort, blood samples were frozen for a median of 10.0 years (range 8.7–12.4 years) and in the PIVUS study, for 2.0 years (range, 0.3–4.0 years) until analysis of Cat-S. The intra-assay coefficient of variation was 7%. To date, there are no established reference values for circulating Cat-S in humans. Soluble TNFR-1 and TNFR-2 were measured as previously described^26^. In ULSAM, we also analyzed urinary Cat-S levels in 513 individuals using the same assay as above. Urine creatinine was analyzed with a modified kinetic Jaffe reaction on an Architect Ci8200^®^ analyzer (Abbott, Abbot Park, IL, USA) and Cat-S/creatinine ratio (in μg/mol) was calculated.

### Statistical Analysis

For statistical analysis IBM SPSS 20 (IBM, Armonk, USA) and Stata 12 (Stata Corp., USA) were used. Continuous data are expressed as mean with standard deviation (SD), categorical variables are reported in absolute numbers and percentages. Normality of distribution for all parameters tested was tested using Shapiro-Wilk-test. Differences of biomarker concentrations between patient groups were evaluated using student’s t-test. Univariate correlations between Cat-S and eGFR, sTNFR1/2 were evaluated using Spearman-rank coefficient in the mouse model and Pearson-correlation coefficient in the human cohorts. Additionally in MCKD, correlations between Cat-S and blood creatinine, BUN and cystatin C were assessed using Pearson-coefficient. To adjust the association between Cat-S (dependent variable) and eGFR/sTNFRs (independent variables) for demographic (age, gender, body-mass-index) and inflammation parameters (CRP) multivariable linear regression modelling was applied in the human cohorts. To assess the association between Cat-S and eGFR, the latter was transformed into a standardized scale. Since sTNFRs (independent variables) were not sufficiently normally distributed, both were transformed into logarithmic scales in the referring linear regression model. In secondary analyses we also investigated the association between urinary Cat-S/creatinine ratio with eGFR using univariate Spearman-Rank correlation coefficient. All reported p-values are two-sided, with a significance level of 0.05. Statistical evaluation was performed similarly in all three cohorts included in the study.

## Results

### Cathepsin-S serum levels increase following the decline of glomerular filtration rate in mice

**Figure 1:**
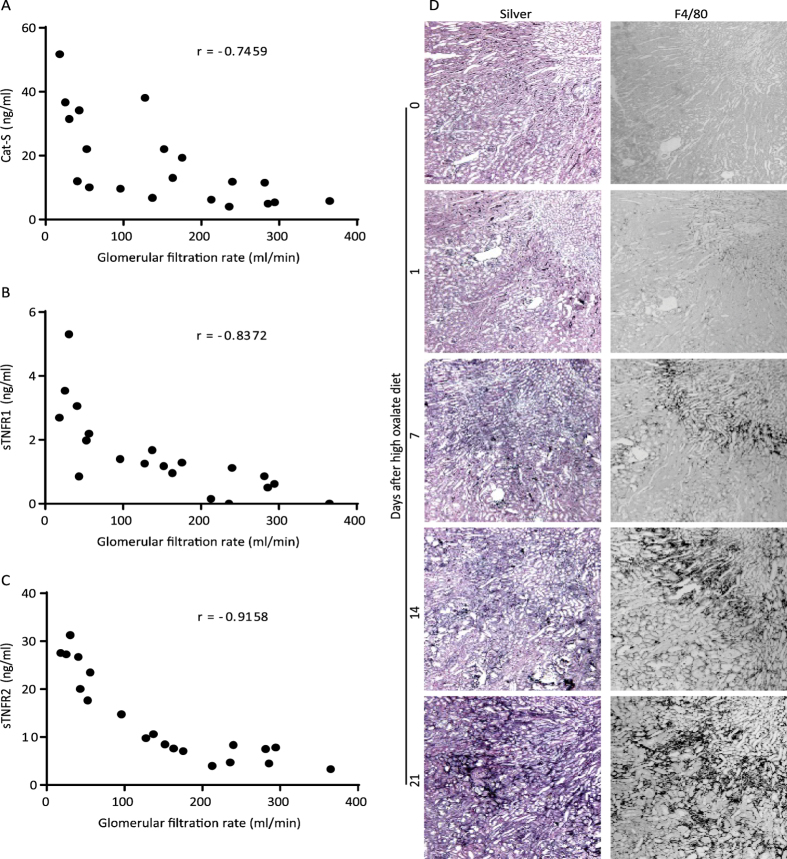
Mouse model of progressive chronic kidney disease: Plasma samples were collected at different time points in oxalate-rich diet fed mice as per the different stages of CKD. We analyzed serum levels of Cat-S (**A**), sTNFR1 (**B**), and sTNFR2 (**C**) plotted with GFR at day 0, 1, 7, 14 and 21. D: Representative images of silver staining and F4/80 positive macrophages (source of Cat-S) in the kidneys of oxalate-rich diet fed mice at different time points. Data expressed as absolute individual values from 5–6 mice each group and Spearman correlation analysis was performed against change in GFR; level of significance for the correlations between GFR and Cat-S, sTNFR1 and sTNFR2 was p < 0.001, respectively.

To address our hypothesis we first studied serum levels of Cat-S in a mouse model of progressive CKD^22^. Feeding mice a sodium oxalate-rich and calcium-depleted diet resulted in a progressive nephrocalcinosis and a continuous decline of GFR, similar to CKD in children with primary hyperoxaluria type 1^27^. Serum samples were obtained at several time points and Cat-S levels were found to increase as CKD progressed with a correlation coefficient of -0.7459 between Cat-S and GFR (p < 0.001, Fig. 1A). Next, we measured serum levels of sTNFR1 and sTNFR2, two established biomarkers of inflammation-related endothelial dysfunction, known to correlate with CKD progression and to predict cardiovascular mortality in CKD^26^. Both sTNFRs showed the same negative correlation with GFR decline (p < 0.001 respectively, Fig. 1B and C). Furthermore the pathophysiologic consequences of oxalate diet-induced CKD could be observed in the mouse kidney morphology using silver staining (Fig. 1D). The histological examination confirmed a progressive tubular atrophy and fibrosis at different time intervals after feeding the oxalate diet, as well as an accumulation of F4/80 positive macrophages during the time course of CKD progression.

### Cathepsin-S serum levels are negatively correlated with estimated glomerular filtration rate in German CKD patients

**Figure 2:**
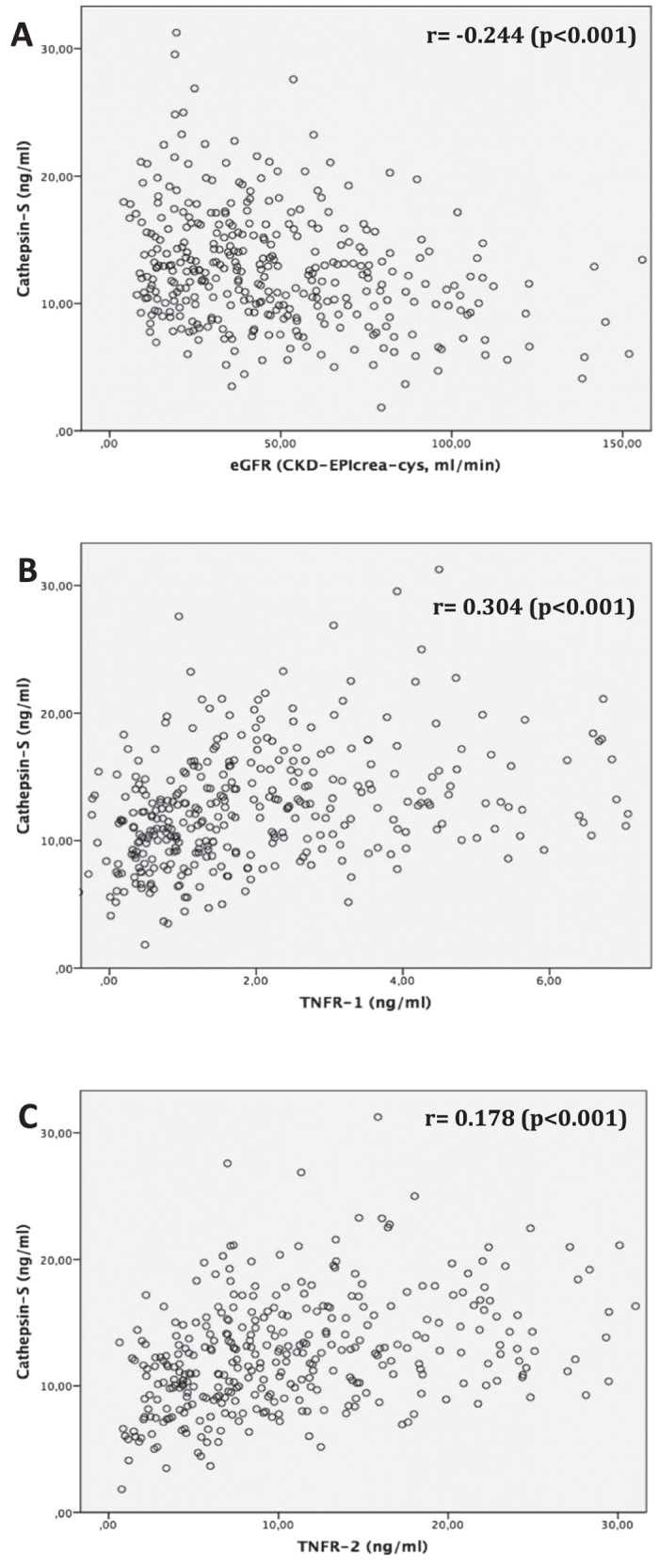
MCKD study; univariate correlation analysis using Pearson-correlation coefficient (r) to assess the association between serum cathepsin-S (in ng/ml) and (**A**) estimated glomerular filtration rate (eGFR CKD-EPI_creatinine-cystatin c_, in ml/min/1.73 m^2^ body surface area); (**B**) serum soluble TNF receptor 1 (TNFR-1, in ng/ml); (**C**) serum soluble TNF receptor 2 (TNFR-2, in ng/ml).

In patients of the Munich CKD cohort (MCKD), plasma Cat-S concentrations progressively increased from CKD I° to CKD V° (Table 1, Fig. 2A). Compared to CKD 0°, the groups CKD III° (p = 0.002), IV° and V° (p < 0.001 respectively) showed significantly higher Cat-S levels, whereas CKD I° (p = 0.674) and CKD II° (p = 0.278) did not differ significantly from CKD 0°. sTNFR1 and sTNFR2 concentrations also increased from CKD I° to CKD V° (Table 1). In univariate analysis including both non-CKD and CKD patients, Cat-S was inversely correlated to eGFR (rho (r) = −0.244, p < 0.001, Fig. 2A) and was positively associated with creatinine (r = 0.167, p = 0.001), BUN (r = 0.166, p = 0.001), cystatin C (r = 0.231, p < 0.001) as well as both sTNFR1 (r = 0.304, p < 0.001, Fig. 2B) and sTNFR2 (r = 0.178, p < 0.001, Fig. 2C). In multivariable linear regression analysis, an increase of one standard deviation in eGFR (37.2 ml/min/1.73 m^2^ BSA) was associated with a decrease of 1.008 ng/ml in Cat-S concentration (p < 0.001, Table 4). Cat-S was also positively associated with (log) sTNFR1 and (log) sTNFR2 in multivariable regression analysis: one unit increase of (log) TNFR1 was associated with an increase of 1.314 ng/ml of Cat-S (p < 0.001, Table 5); an increase of one unit of (log) TNFR2 was associated with an increase of 1.976 ng/ml of Cat-S (p < 0.001, Table 5).

### Cathepsin-S serum levels are negatively correlated with glomerular filtration rate in two Swedish population cohorts

**Figure 3:**
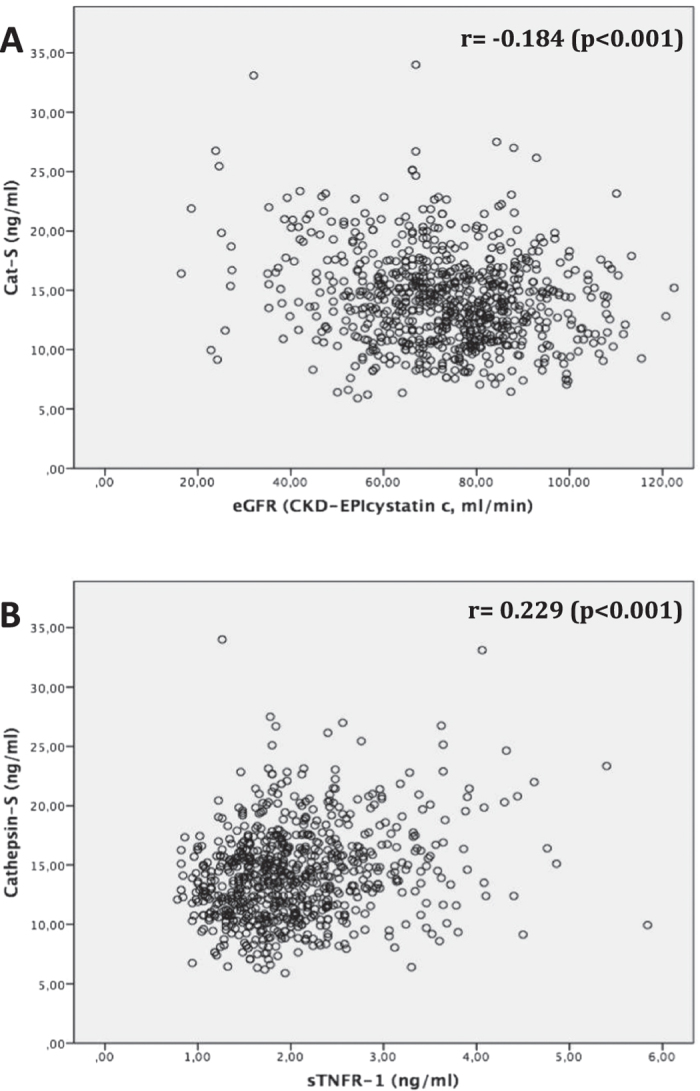
ULSAM study; univariate correlation analysis using Pearson-correlation coefficient (r) to assess the association between serum cathepsin-S (in ng/ml) and (**A**) estimated glomerular filtration rate (eGFR CKD-EPI_cystatin c_, in ml/min/1.73 m^2^ body surface area); (**B**) serum soluble TNF receptor 1 (TNFR-1, in ng/ml).

**Figure 4:**
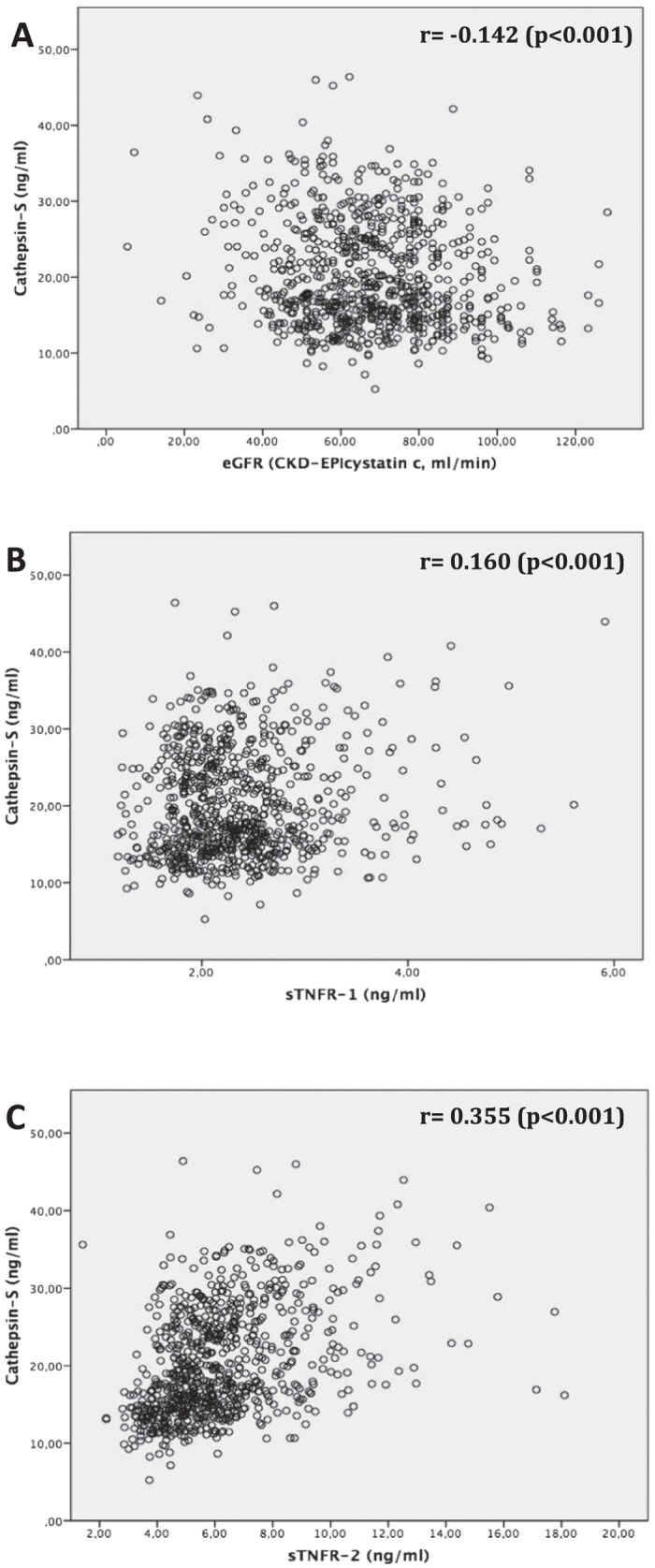
PIVUS study; univariate correlation analysis using Pearson-correlation coefficient (r) to assess the association between serum cathepsin-S (in ng/ml) and (**A**) estimated glomerular filtration rate (eGFR CKD-EPI_cystatin c_, in ml/min/1.73 m^2^ body surface area); (**B**) serum soluble TNF receptor 1 (TNFR-1, in ng/ml); (**C**) serum soluble TNF receptor 2 (TNFR-2, in ng/ml).

To validate the data from the MCKD cohort using unrelated and unselected population cohorts, we analyzed two population-based samples, ULSAM and PIVUS from the Uppsala region in Sweden. Similarly to MCKD, Cat-S concentrations gradually increased with deterioration of kidney function in both cohorts (Tables 2 and 3). In univariate analysis, serum Cat-S was negatively correlated with eGFR in both cohorts (p < 0.001 respectively, Figs 3A and 4A). Analogous to MCKD, Cat-S was positively associated with sTNFR1 in both ULSAM (p < 0.001, Fig. 3B) and PIVUS (p < 0.001, Fig. 4B). sTNFR2 was only available in PIVUS, in which it also positively associated with Cat-S (p < 0.001, Fig. 4C). In multivariable linear regression analysis, an increase of one standard deviation of eGFR (±17.1 ml/min/1.73 m^2^ BSA in ULSAM, ±19.3 ml/min/1.73 m^2^ BSA in PIVUS) resulted in a 0.664 ng/ml decrease of Cat-S serum concentrations (p < 0.001, Table 4) in ULSAM and a 1.076 ng/ml decrease (p < 0.001, Table 4) in PIVUS. Cat-S was also significantly associated with (log) sTNFR1 in both ULSAM and PIVUS: a one-unit increase of (log) sTNF1 was associated with a Cat-S increase of 2.474 ng/ml in ULSAM and 4.034 ng/ml in PIVUS (p < 0.001 respectively, Table 5). In PIVUS, Cat-S and (log) sTNFR2 showed a significant association, with a Cat-S increase of 8.935 ng/ml per unit increase of (log) sTNF2 (p < 0.001, Table 5). There was no association between urinary Cat-S/creatinine ratio with eGFR (p = 0.465, Supplementary Figure 1).

Taken together, we conclude that Cat-S serum levels show an negative correlation with renal function as defined by measured (mice) or estimated (human) GFR. This inverse correlation is identical to those of sTNFR1 and sTNFR2, two validated markers of systemic inflammation-related endothelial dysfunction.

## Discussion

We hypothesized that the progression of CKD and its related systemic inflammatory state is associated with increasing levels of circulating Cat-S, a protease involved in the pathogenesis of cardiovascular disease and a potential therapeutic target to attenuate atherosclerosis in CKD^19^. Our approach to address this issue included experiments in which we induced CKD in mice, and cross-sectional analyses of three European human cohorts, one of selected CKD patients (MCKD) and two population-based cohorts (PIVUS, ULSAM) of elderly individuals. Our data confirm the hypothesis and reveal a consistent negative correlation between Cat-S serum levels and measured GFR (mice) or estimated GFR (humans). In addition, we found a positive correlation of Cat-S levels with markers of glomerular function (blood creatinine, BUN and cystatin C) as well as with two established markers of systemic inflammation in CKD patients, sTNFR1 and sTNFR2^26^. This shows a robust association of Cat-S levels with different markers of glomerular function and inflammation.

These results establish the impact of renal function on Cat-S concentrations in the circulation. We recently reported that Cat-S is filtered across the glomerular filtration barrier^5^. Because proximal tubular cells of mouse and human kidneys contain Cat-S protein but no RNA transcripts, intrarenal Cat-S mRNA (and protein) expression largely originates from infiltrating CD68+ macrophages that (unlike tubular epithelial cells) do not express sufficient amounts of the intrinsic Cat-S inhibitor cystatin-C^5^. We therefore assume that in CKD circulating Cat-S levels are largely produced from activated circulating monocytes but not locally in renal tissue^4^. This hypothesis is further strengthened by the fact that we could not detect a significant association between urinary Cat-S/creatinine ratio and eGFR in the ULSAM cohort (Supplementary Figure 1). Furthermore, circulating Cat-S was not associated with proteinuria/albuminuria in all three cohorts (Supplementary Table 1). Both increased production and a decline of glomerular filtration surface, as it occurs along nephron loss during CKD, may reduce the clearance of Cat-S from the circulation, and therefore, increase circulating Cat-S levels. In fact, Cat-S has a similar molecular weight to several markers of glomerular filtration such as cystatin C, beta-trace protein, and beta2-microglobulin. This molecular weight similarity suggests that Cat-S may be freely filtered through the glomeruli and thus provide a potential marker of GFR.

Markers of systemic inflammation, such as urinary monocyte chemotactic protein-1, tumor necrosis factor-alpha (TNF-α), and interleukin-6 (IL-6) have been shown to correlate with the severity of CKD^28^,^29^. In addition, inflammatory markers sTNFR1 and sTNFR2 have been associated with CKD^26^. The latter markers are not only biomarkers of systemic inflammation but also predict cardiovascular morbidity and mortality^13^. Both TNFRs are shed mainly by activated endothelial cells and are therefore considered to represent markers of inflammation-related endothelial dysfunction^30^. Other markers of endothelial dysfunction, such as asymmetric dimethylarginine (ADMA), L-arginine, soluble vascular adhesion molecule-1 (sVCAM-1) and soluble E-selectin (sE-selectin) have also been associated with CKD^31^, demonstrating that endothelial dysfunction contributes to cardiovascular morbidity/mortality in CKD patients. We have recently shown that Cat-S is an upstream inducer of inflammation-related endothelial dysfunction by cleaving the extracellular domain of the protease-activated receptor (PAR)-2 on the luminal membranes of vascular endothelial cells^5^. As a working hypothesis, any stimulus that activates monocytes and neutrophils in the circulation, as may occur during extra-corporal circulation during hemodialysis or by bacterial endotoxin leakage from the intestinal tract^32^, may induce Cat-S release, which activates endothelial cells to release sTNFR1 and sTNFR2. In line with this hypothesis Cat-S has been previously shown to be associated with the sTNFRs in hemodialysis patients, a highly inflammatory milieu^33^. The vasculopathic effects of Cat-S also have a direct role in promoting cardiovascular complications, such as the processes of atherogenesis and vascular wall degeneration, including aneurysm formation and media calcification^6^,^7^,^8^,^9^,^10^,^11^,^12^,^18^,^19^,^34^.

Interestingly, we observed that Cat-S and sTNFR1/2 concentrations differed between the three human cohorts in groups with similar eGFR (e.g. CKD III°). Therefore we wondered if other demographic/laboratory parameters besides kidney function might influence their blood concentrations. We reran an exemplary multivariable linear regression analysis focusing on parameters possibly influencing Cat-S in MCKD. We set Cat-S as the dependent variable and added independent variables as follows: eGFR, gender, age, BMI, total serum protein, serum CRP and prevalence of diabetes mellitus, arterial hypertension, vascular disease and tumor disease. In this analysis, besides eGFR only gender was weakly associated with Cat-S (p = 0.043), otherwise no significant data was obtained. Since the clinical samples were similar, we surmise that methodological rather than clinical circumstances lead to these findings. In particular, the sample material may have an influence on the results since plasma was used in MCKD and serum in PIVUS/ULSAM. Furthermore, samples were stored for a much longer period of time in PIVUS/ULSAM. Currently there are no reference values for Cat-S or sTNFR 1/2 in human material, and it would be important to determine in the future whether different value ranges exist for different sample materials and storage conditions.

Our study has several strengths, including the use of both experimental and clinical data and the validation of results in independent clinical cohorts with a wide range of glomerular filtration rate. One limitation is that statistical analyses were not adjusted for multiple testing. However, the consistency of results from three different cohorts and a mouse model would argue against spurious association as an explanation of our findings. Other limitations include the lack of longitudinal data in the human cohorts and the use of different sample material in the three human cohorts, possibly limiting the comparability of the results obtained in the three cohorts. It is acknowledged that our data are cross-sectional in nature and only reflect the association between Cat-S and kidney function, with no outcome data provided. Hence, no clinical consequences can be drawn from our findings. However, the association of circulatory Cat-S with outcomes in CKD patients should be evaluated in future epidemiological studies. Lastly, additional markers of endothelial dysfunction and systemic inflammation that have been shown to be associated with CKD could not be measured in our cohorts, and might have been compared with Cat-S to validate the proinflammatory status.

In summary, Cat-S, a cysteine protease released by activated immune cells, is consistently found at increased levels that correlate with the progression of CKD in mice and humans. It is tempting to speculate that Cat-S is a causative factor in this association, and a mediator of CKD-related cardiovascular morbidity and mortality. As pharmacological Cat-S inhibition has been shown to prevent macrovascular and microvascular disease in experimental animal models, there appears to be a valid scientific rationale to study whether Cat-S is associated with adverse outcomes in CKD and whether its inhibition can reduce cardiovascular morbidity and mortality in CKD patients.

## Additional Information

**How to cite this article:** Steubl, D. *et al*. Circulating cathepsin-S levels correlate with GFR decline and sTNFR1 and sTNFR2 levels in mice and humans. *Sci. Rep.* **7**, 43538; doi: 10.1038/srep43538 (2017).

**Publisher's note:** Springer Nature remains neutral with regard to jurisdictional claims in published maps and institutional affiliations.

## Supplementary Material

Supplementary Information

## Figures and Tables

**Table 1:** Demographic characteristics and laboratory parameters in MCKD cohort.

MCKD	Total (n = 421)	CKD 0° (n = 69, 16.4%)	CKD I° (n = 37, 8.8%)	CKD II° (n = 68, 16.2%)	CKD III° (n = 126, 29.9%)	CKD IV° (n = 80, 19.0%)	CKD V° (n = 41, 9.7%)
*Age (years, SD*)	56.8 (±16.4)	49.1(±15.1)	41.6 (±14.9)	52.3 (±14.3)	63.8 (±13.6)	62.2 (±16.2)	59.1 (±14.9)
*Gender (male, %*)	246 (58.4)	29 (42.0)	19 (51.4)	39 (57.4)	80 (63.5)	52 (65.0)	27 (65.9)
*BMI (kg*/*m*^*2*^, *SD*)	26.9 (±5.7)	25.0 ± 6.0	26.1 (±5.6)	26.6 (±5.6)	26.9 (±5.0)	27.6 (±6.0)	28.5 (±6.1)
*Diabetes mellitus (n, %*)	96 (22.8)	5 (7.2)	3 (8.1)	8 (11.8)	30 (23.8)	29 (36.3)	21 (29.3)
*Antidiabetic medication (n, %*)	72 (17.1)	5 (7.2)	3 (8.1)	5 (7.4)	28 (22.2)	22 (27.5)	9 (22.0)
*Arterial hypertension (n, %*)	277 (65.8)	13 (18.8)	21 (56.8)	39 (57.4)	99 (78.6)	66 (82.5)	39 (95.1)
*Systolic blood pressure (mmHg, SD*)	137.5 (±20.1)	135.7 (±20.4)	137.1 ± 20.6	133.5 (±17.8)	136.1 (±19.9)	140.9 (±18.1)	144.0 (±25.6)
*Diastolic blood pressure (mmHg, SD*)	82.8 (±12.3)	83.1 (±10.9)	86.4 ± 11.8	82.7 (±11.7)	82.7 (±12.4)	81.1 (±13.5)	82.8 (±12.0)
*Antihypertensive medication (n*)	320	13	26	55	114	72	40
*Cardiovascular disease (n, %*)	56 (13.3)	2 (2.9)	2 (5.4)	4 (5.9)	22 (17.5)	18 (22.5)	8 (19.5)
*Creatinine (mg*/*dl, SD*)	2.0 (±1.7)	0.9 (±0.2)	1.0 (±0.3)	1.3 (±0.3)	1.7 (±0.4)	2.8 (±0.8)	6.0 (±2.6)
*Cystatin C (mg*/*l, SD*)	1.53 (±0.96)	0.65 (±0.17)	0.58 (±0.18)	0.91 (0.16)	1.44 (±0.29)	2.41 (±0.50)	3.52 ±0.63)
*BUN (mg*/*dl, SD*)	29.8 (±17.9)	13.5 (±3.8)	14.8 (±4.8)	19.3 (±5.2)	27.3 (±9.2)	43.1 (±12.7)	63.9 (±16.8)
*eGFR (ml*/*min*/*1.73 m*^*2*^ *BSA, SD*)	57.9 (±37.2)	108.2 (±21.8)	113.1 (±20.5)	73.1 (±8.7)	43.6 (±8.4)	22.6 (±4.6)	11.5 (±3.0)
*Cathepsin*-*S*, (*ng*/*ml, SD*)	12.5 (±5.0)	10.8 (±4.1)	11.2 (±8.5)	11.6 (±4.1)	12.9 (±4.4)	14.0 (±5.3)	13.5 (±3.8)
*sTNFR1 (ng*/*ml, SD*)	1.94 (±2.07)	0.67 (±0.62)	0.52 (±0.59)	0.83 (±0.57)	1.51 (±0.93)	3.44 (±1.74)	5.59 (±3.03)
*sTNFR2 (ng*/*ml, SD*)	11.6 (±16.7)	7.1 (±23.3)	4.5 (±2.8)	5.3 (±3.1)	10.3 (±13.8)	20.2 (±21.0)	23.4 (±7.9)
*CRP (mg*/*dl, SD*)	0.7 (±1.6)	0.3 (±0.4)	0.2 (±0.3)	0.5 (±1.0)	0.7 (±1.4)	1.1 (±2.1)	1.4 (±2.8)
*Protein*/*creatinine ratio (mg*/*g, SD*)	872.6 (±1697.9)	59.6 (±63.1)	827.3 (±1523.1)	474.2 (±809.7)	643.3 (±1164.7)	1875.4 (±2791.4)	2143.3 (±1609.9)
***Underlying disease***							
*None (n, %*)	69 (16.4)	69 (100)	—	—	—	—	—
*Diabetic nephropathy (n, %*)	26 (6.2)	—	0 (0)	1 (1.5)	10 (7.9)	9 (11.3)	6 (14.6)
*Hypertensive nephropathy (n, %*)	58 (13.8)	—	4 (10.8)	7 (10.3)	16 (12.7)	19 (23.8)	12 (29.3)
*Glomerulonephritis (n, %*)	112 (26.6)	—	17 (45.9)	26 (38.2)	39 (31.0)	22 (27.5)	8 (19.5)
*Polycystic kidney disease (n, %*)	15 (3.6)	—	4 (10.8)	3 (4.4)	4 (3.2)	2 (2.5)	2 (4.9)
*Postrenal failure (n, %*)	29 (6.9)	—	1 (2.7)	9 (13.2)	13 (10.3)	4 (5.0)	2 (4.9)
*Others*/*unknown (n, %*)	112 (26.6)	—	11 (29.7)	22 (32.4)	44 (34.9)	24 (30.0)	11 (26.8)

Data are presented as mean ± standard deviation or as absolute numbers (n) with percentage (%) in brackets; CKD = chronic kidney disease; BMI = body mass index; BUN = blood-urea nitrogen; eGFR calculated from CKD-EPI_creatinine-cystatin c_ formula; BSA = body surface area; sTNFR = soluble tumor-necrosis-factor receptor; CRP = c-reactive protein; protein/creatinine ratio in urine; antihypertensive medication also includes ACE-inhibitors/AT1-receptor antagonists, which also might have been used for solely antihypertensive medication; therefore, the number of patients with antihyperrtensive medication exceeds the number of patients with arterial hypertension in CKD groups.

**Table 2:** Demographic characteristics and laboratory parameters in ULSAM cohort (100% male).

ULSAM	Total (n = 749)	CKD 0° (n = 85, 11.4%)	CKD I° (n = 20, 2.7%)	CKD II° (n = 491, 65.6%)	CKD III° (n = 142, 19.0%)	CKD IV° (n = 11, 1.5%)	CKD V° (n = 0)
*Age (years, SD*)	77.6 (±0.78)	77.4 (±0.83)	77.6 (±0.79)	77.6 (±0.78)	77.6 (±0.68)	77.8 (±0.74)	—
*BMI (kg*/*m*^*2*^, *SD*)	26.3 (±3.5)	25.9 ± 2.8	26.1 (±5.6)	26.2 (±3.39)	27.0 (±4.0)	26.8 (±4.3)	—
*Diabetes mellitus (n, %*)	82 (10.9)	13 (15.3)	3 (15.0)	1 (0.2)	61 (43.0)	4 (3)	—
*Antidiabetic medication (n, %*)	66 (8.8)	7 (8.2)	1 (5.0)	37 (7.5)	19 (13.4)	2 (18.2)	—
*Arterial hypertension (n, %*)	332 (44.3)	28 (32.9)	7 (35.0)	193 (39.3)	96 (67.6)	8 (72.3)	—
*Systolic blood pressure (mmHg, SD*)	150 (±21)	147 (±21)	149 ± 20	151 (±20)	150 (±21.4)	161 (±31)	—
*Diastolic blood pressure (mmHg, SD*)	81.3 (±9.8)	79.3 (±9.1)	81.0 ± 13.3	81.7 (±9.4)	81.1 (±10.8)	80.7 (±7.7)	—
*Antihypertensive medication (n, %*)	307 (41.0)	27 (31.8)	6 (30.0)	174 (35.4)	92 (64.8)	8 (72.7)	—
*Cardiovascular disease (n, %*)	204 (27.2)	17 (20.0)	1 (5.0)	118 (24.0)	62 (43.7)	6 (54.5)	—
*Lipid*-*lowering therapy (n, %*)	127 (17.0)	17 (20.0)	2 (10.0)	72 (14.7)	33 (23.2)	3 (27.3)	—
*LDL (mmol*/*l, SD*)	3.5 (±0.9)	3.6 (±0.9)	3.7 (±0.7)	3.5 (±0.9)	3.4 (±0.9)	3.3 (±1.0)	—
*HDL (mmol*/*l, SD*)	1.3 (±0.3)	1.4 (±0.4)	1.5 (±0.3)	1.3 (±0.3)	1.2 (±0.3)	1.1 (±0.2)	—
*Cystatin C (mg*/*l, SD*)	1.09 (±0.28)	0.82 (±0.05)	0.83 (±0.04)	1.03 (±0.09)	1.41 (±0.17)	2.58 (±0.28)	—
*eGFR (ml*/*min*/*1.73 m*^*2*^ *BSA, SD*)	73.3 (±17.1)	100.2 (±9.1)	98.0 (±6.9)	75.1 (±8.1)	51.2 (±7.0)	23.9 (±3.5)	—
*Cathepsin*-*S*, (*ng*/*ml, SD*)	14.3 (±4.0)	13.4 (±3.5)	15.1 (±3.2)	14.1 (±3.9)	15.1 (±4.4)	17.4 (±5.9)	—
*sTNFR1 (ng*/*ml, SD*)	2.07 (±0.81)	1.61 (±0.49)	1.16 (±0.60)	2.0 (±0.64)	1.51 (±0.80)	5.06 (±1.9)	—
*CRP (mg*/*dl*)	0.4 (±0.7)	0.3 (±0.5)	0.4 (±0.7)	0.4 (±0.5)	0.6 (±1.2)	0.5 (±0.2)	—
Albumin/creatinine ratio (mg/g, SD)	38.9 (±169.00)	6.8 (±5.5)	50.0 (±36.6)	26.4 (±93.3)	45.2 (±100.3)	836.0 (±1001.4)	—

Data are presented as mean ± standard deviation or as absolute numbers (n) with percentage (%) in brackets; CKD = chronic kidney disease; BMI = body mass index; LDL = low-density lipoprotein; HDL = high-density lipoprotein; eGFR calculated from cystatin c formula; BSA = body surface area; sTNFR = soluble tumor-necrosis-factor receptor; CRP = c-reactive protein; Albumin/creatinine ratio in urine.

**Table 3:** Demographic characteristics and laboratory parameters in PIVUS cohort.

PIVUS	Total (n = 804)	CKD 0° (n = 69, 8.6%)	CKD I° (n = 18, 2.2%)	CKD II° (n = 443, 55%)	CKD III° (n = 261, 32%)	CKD IV° (n = 13, 1.6%)	CKD V° (n = 0, 0%)
*Age (years, SD*)	75.3 (±0.19)	75.3 (±0.18)	75.3 (±0.19)	75.3 (±0.18)	75.3 (±0.19)	75.3 (±0.23)	—
*Gender (male, %*)	408 (50.8)	47 (68.1)	7 (38.9)	245 (55.3)	105 (40.2)	4 (30.7)	—
*BMI (kg*/*m*^*2*^, *SD*)	26.8 (±4.3)	25.4 ± 3.5	23.6 (±3.0)	26.5 (±4.1)	28.0 (±4.6)	27.7 (±5.8)	—
*Diabetes mellitus (n, %*)	93 (11.6)	7 (10.1)	1 (5.5)	41 (9.3)	40 (15.3)	4 (30.7)	—
*Antidiabetic medication (n, %*)	72 (9.0)	4 (5.8)	1 (5.6)	32 (7.2)	32 (12.3)	3 (23.1)	—
*Arterial hypertension (n, %*)	654 (81.3)	47 (68.2)	16 (88.9)	360 (81.3)	218 (83.5)	13 (100)	—
*Systolic blood pressure (mmHg, SD*)	149 (±19)	147 (±18.6)	155 (± 21.2)	148 (±18.6)	149 (±20.7)	150 (±21.9)	—
*Diastolic blood pressure (mmHg, SD*)	75.9 (±9.4)	73.5 (±10.2)	80.2 ± 9.8	76.2 (±9.0)	75.9 (±9.5)	72.8 (±14.4)	—
*Antihypertensive medication (n*)	389 (48.4)	22 (31.9)	7 (38.9)	187 (42.2)	162 (62.1)	11 (84.6)	—
*Cardiovascular disease (n, %*)	68 (8.5)	3 (4.3)	2 (11.1)	18 (4.1)	40 (15.3)	5 (38.5)	—
*Lipid*-*lowering therapy (n, %*)	206 (25.6)	19 (27.5)	4 (22.2)	104 (23.5)	74 (28.4)	5 (38.5)	—
*LDL (mmol*/*l, SD*)	3.4 (±0.9)	3.5 (±0.9)	3.6 (±1.4)	3.5 (±0.9)	3.1 (±0.9)	3.2 (±0.8)	—
*HDL (mmol*/*l, SD*)	1.5 (±0.5)	1.8 (±0.5)	1.5 (±0.4)	1.5 (±0.4)	1.3 (±0.4)	1.3 (±0.5)	—
*Cystatin C (mg*/*l, SD*)	0.92 (±0.18)	0.73 (±0.08)	0.74 (±0.09)	0.87 (±0.09)	1.05 (±0.16)	1.52 (±0.40)	—
*eGFR (ml*/*min*/*1.73 m*^*2*^ *BSA, SD*)	67.8 (±19.3)	102.2 (±20.3)	102.6 (±10.7)	72.9 (±8.0)	50.1 (±7.2)	21.0 (±7.5)	—
*Cathepsin*-*S*, (*ng*/*ml, SD*)	20.4 (±7.4)	17.9 (±5.6)	18.6 (±6.7)	20.3 (±7.1)	21.1 (±7.9)	25.0 (±11.2)	—
*sTNFR1 (ng*/*ml, SD*)	2.46 (±1.3)	1.81 (±0.35)	1.82 (±0.37)	2.18 (±0.44)	2.93 (±1.43)	3.44 (±1.74)	—
*sTNFR2 (ng*/*ml, SD*)	6.33 (±2.89)	4.68 (±1.41)	6.21 (±7.34)	5.71 (±1.66)	7.42 (±2.49)	6.76 (±5.49)	—
*CRP (mg*/*dl*)	0.2 (±0.3)	0.2 (±0.3)	0.2 (±0.3)	0.2 (±0.4)	0.2 (±0.3)	0.2 (±0.1)	—
Albumin/creatinine ratio (mg/g, SD)	54.6 (±255.2)	11.3 (±6.0)	111.8 (±115.3)	33.1 (±139.0)	90.2 (±398.6)	294.8 (±356.4)	—

Data are presented as mean ± standard deviation or as absolute numbers (n) with percentage (%) in brackets; CKD = chronic kidney disease; BMI = body mass index; LDL = low-density lipoprotein; HDL = high-density lipoprotein; eGFR calculated from cystatin c formula; BSA = body surface area; sTNFR = soluble tumor-necrosis-factor receptor; CRP = c-reactive protein;

**Table 4:** Multivariable linear regression models showing the association between standard deviation increments in eGFR (independent variable, in ml/min/1.73 m^2^ body surface area (BSA)) and cathepsin-S (dependent variable, in ng/ml) in all three study cohorts.

Study group	regression coefficient B	95% confidence intervals	p-value
*MCKD*	−1.008	−1.576–(−0.439)	<0.001
*ULSAM*	−0.664	−0.952–(−0.375)	<0.001
*PIVUS*	−1.076	−1.604–(−0.547)	<0.001

Multivariable analysis adjusted for age, gender, body-mass-index and c-reactive protein; the regression coefficient B describes the change of cathepsin-S (in ng/ml) per change of one standard deviation (SD) of eGFR in every cohort (SD in MCKD 37.2 ml/min/1.73 m2 BSA, in ULSAM 17.1 ml/min/1.73 m2 BSA, in PIVUS 19.3 ml/min/1.73 m2 BSA).

**Table 5:** Multivariable linear regression models showing the association between (log) sTNFR-1/-2 (independent variable, in ng/ml) and cathepsin-S (dependent variable, in ng/ml) in all three study cohorts.

Study group/Biomarker	regression coefficient B	95% confidence interval	p-value
***MCKD***			
Cathepsin-S vs sTNFR-1	1.314	0.833–1.794	<0.001
*Cathepsin*-*S vs sTNFR*-*2*	1.976	1.437–2.514	<0.001
***ULSAM***			
*Cathepsin*-*S vs sTNFR*-*1*	2.474	1.654–3.293	<0.001
***PIVUS***			
*Cathepsin*-*S vs sTNFR*-*1*	4.034	2.297–5.772	<0.001
*Cathepsin*-*S vs sTNFR*-*2*	8.935	7.548–10.323	<0.001

Multivariable analysis adjusted for age, gender, body-mass-index, and c-reactive protein; sTNFR-2 not available in ULSAM; the regression coefficient B describes the change of cathepsin-S (in ng/ml) per change of (log) sTNFR-1/2.
