# Supplementary material for: Circulating cathepsin-S levels correlate with GFR decline and sTNFR1 and sTNFR2 levels in mice and humans
Source: Sci Rep. 2017 Feb 27;7:43538. doi: 10.1038/srep43538 (PMC5327444; doi:10.1038/srep43538)
Supplement: Supplementary Information [file srep43538-s1.docx]

**Circulating cathepsin-S levels correlate with GFR decline and sTNFR1 and sTNFR2 levels in mice and humans**

Dominik Steubl^1^*, Santhosh V. Kumar^2^*, Maia Tato^2^, Shrikant R. Mulay^2^, Anders Larsson^3^, Lars Lind^3^, Ulf Risérus^4^, Lutz Renders^1^, Uwe Heemann^1^, Axel C Carlsson^3,5^, Johan Ärnlöv^3,6^, Hans-Joachim Anders^2^

1 Abteilung für Nephrologie, Klinikum rechts der Isar, Technische Universität München, Munich, Germany

2 Medizinische Klinik und Poliklinik IV, Renal Division, Klinikum der Universität München, Campus Innenstadt, München, Germany

3 Department of Medical Sciences, Uppsala University, Uppsala, Sweden

4 Departments of Public Health and Caring Sciences/Clinical Nutrition, Uppsala University, Uppsala, Sweden

5 Division of Family Medicine, Department of Neurobiology, Care Sciences and Society, Karolinska Institutet, Huddinge, Sweden

6 School of Health and Social Studies, Dalarna University, Falun, Sweden

* Equal contribution

| **Study group / Biomarker** | **regression coefficient B** | **95% confidence interval** | **p-value** |
| --- | --- | --- | --- |
| ***MCKD*** |  |  |  |
| *Cathepsin-S vs proteinuria* | *-0.029* | *(-0.625)-0.568* | *0.925* |
| ***ULSAM*** |  |  |  |
| *Cathepsin-S vs albuminuria* | *0.14* | *(-0.17)-0.45* | *0.380* |
| ***PIVUS*** |  |  |  |
| *Cathepsin-S vs albuminuria* | *0.18* | *(-0.35)-0.71* | *0.510* |

**Supplementary table 1:** Multivariable linear regression models showing the association between (log) proteinuria (MCKD, in mg/g creatinine) and (log) albuminuria (ULSAM/PIVUS, independent variable, in mg/g creatinine) and cathepsin-S (dependent variable, in ng/ml) in all three study cohorts

Multivariable analysis adjusted for age, gender, body-mass-index, c-reactive protein and estimated glomerular filtration rate; proteinuria/albuminuria in standardized scale; regression coefficient B expresses the change in cathepsin-S per 1 standard deviation increase of proteinuria/albuminuria;

**Supplementary figure 1**


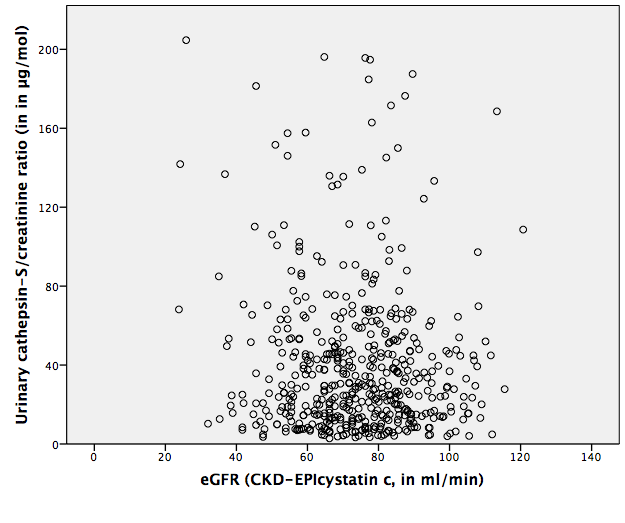


**r= -0.032 (p=0.465)**

**Supplementary figure 1** ULSAM study; univariate correlation analysis using Spearman-Rank correlation coefficient (r) to assess the association between urinary cathepsin-S/creatinine ratio (in μg/mol) and estimated glomerular filtration rate (eGFR CKD-EPI_cystatin c_, in ml/min/1.73 m^2^ body surface area);
